# Menstrual dignity and menstrual experiences among riverside adolescents in the Brazilian Amazon: participatory development of an educational technology using Paulo Freire’s research itinerary

**DOI:** 10.1371/journal.pone.0354168

**Published:** 2026-07-24

**Authors:** Amanda Guimarães Cunha, Marta Lenise do Prado, Elizabeth Teixeira, Cláudio Claudino da Silva Filho, Mary Elizabeth de Santana, Rubenilson Caldas Valois, George Pinheiro Carvalho, Lucrecia Aline Cabral Formigosa, Haroldo Gonçalves de Jesus, Reinaldo de Souza Guimarães, Gisele Maria Cardoso da Silva, Marinara de Nazaré Araújo Lobato, Bruna Eduarda Belo Gaia, Lucas Geovane dos Santos Rodrigues, Marcia Helena Machado Nascimento

**Affiliations:** 1 Department of Nursing, State University of Pará, Belém, Pará, Brazil; 2 Department of Nursing, Federal University of Santa Catarina, Florianópolis, Santa Catarina, Brazil; 3 Department of Nursing, Federal University of Fronteira Sul, Chapecó, Santa Catarina, Brazil; Cranfield University, UNITED KINGDOM OF GREAT BRITAIN AND NORTHERN IRELAND

## Abstract

**Objective:**

To understand menstrual experiences and perceptions of menstrual dignity among riverside adolescent girls in the Brazilian Amazon in order to support the participatory co-creation of an educational technology using Paulo Freire’s research itinerary.

**Methods:**

Technology development research, with a qualitative approach and participatory interface, grounded in Paulo Freire’s research itinerary, composed of three stages: thematic investigation, coding/decoding and critical unveiling. The study was conducted in a public school located on Caratateua Island, Pará, Brazil, with the participation of 10 adolescents, and the investigation was operationalized through Culture Circles. Data were collected through audio recordings and field diary notes and analyzed using inductive thematic analysis aligned with Freirean stages, and the findings supported the participatory co-creation of an educational technology in the format of a comic book.

**Results:**

Three main thematic categories emerged: (1) menstruation as emotional and daily life disruption, (2) silence and limited dialogue about menstrual experiences, and (3) sociocultural beliefs influencing menstrual care practices. These findings informed the development of a culturally contextualized comic book addressing menstrual knowledge, self-care, and menstrual dignity.

**Conclusion:**

The findings highlight the importance of culturally grounded educational approaches for menstrual health. The participatory co-creation process enabled the development of an educational resource aligned with adolescents’ lived experiences and may support future health education strategies and research in menstrual health promotion.

## Introduction

The United Nations (UN) declares that menstrual dignity is a matter of public health and human rights, essential for ensuring the right to bodily autonomy and self-determination for people who menstruate, including access to menstrual products and adequate hygiene conditions [1,2].

Menstrual dignity encompasses access to information about menstrual health, the availability of clean and private facilities for managing menstruation, and freedom from shame and embarrassment associated with menstruation [3]. Physiologically, menstruation is defined as the shedding of blood from the endometrium through the vaginal canal, occurring when blood levels of follicle-stimulating hormone (FSH) and luteinizing hormone (LH) decrease [4].

Socially, menstruation is a process related not only to women, but also to their social and cultural groups [3]. It is surrounded by rituals and becomes a significant event. According to sociocultural aspects, each social group assigns its own meaning and interpretation to menstruation, which marks a woman’s life as a period of transition through two distinct stages. Some cultures, for instance, attribute a high social and communal value to menstruation, celebrating it with rituals such as festivities for girls experiencing menarche. Others, however, impose social exclusion on women during this time, treating it as a form of taboo — for example, in many fishing communities along the Amazon coast, menstruating women are not allowed to participate in fishing activities [5,6].

Menstrual poverty refers to limited access to hygiene products, adequate sanitation infrastructure, information, and supportive environments that allow women to manage menstruation safely and with dignity. This condition affects millions of girls and women worldwide and may contribute to school absenteeism, stigma, and social exclusion. Consequently, menstrual health is closely linked to human dignity and to several Sustainable Development Goals established by the UN, particularly those related to health, education, gender equality, and access to water and sanitation [2,7,8].

In Brazil, menstrual poverty disproportionately affects socially vulnerable regions, including the Northern region, where limited access to menstrual products and sanitation infrastructure remains a significant challenge. According to data from the study “The Impact of Menstrual Poverty in Brazil,” conducted by the brand Always in partnership with the research platform Toluna, 36% of women in the region have experienced periods during which they were unable to afford menstrual hygiene products [9].

From this perspective, understanding menstrual health care also requires considering the beliefs and traditions that shape menstrual experiences in different sociocultural contexts. In the Brazilian Amazon, many traditional communities—such as riverine populations living in rural and coastal areas—maintain cultural practices and knowledge systems that influence perceptions and management of menstruation. Rethinking care and popular care practices therefore becomes essential for understanding how menstrual health is experienced and addressed within these communities [10].

In this regard, menstruation and the social expectations associated with it reveal many of the challenges still experienced by adolescent girls in different sociocultural contexts. In traditional communities of the Brazilian Amazon, cultural norms and beliefs surrounding menstruation may shape how girls experience their bodies and participate in everyday social spaces. These dynamics can influence their autonomy and affect how freely they engage in school, community and family life [11].

Paulo Freire was a Brazilian educator and philosopher widely recognized for his contributions to critical pedagogy, which emphasizes dialogue, participation, and the co-construction of knowledge. From the Freire’s perspective, understanding the learners’ reality and context is essential for them to make sense of their experiences and achieve liberation. The exchange and sharing of experiences and information between learner and educator occur through a process that enables the construction of knowledge that is meaningful to the learner. In this way, the learner becomes not merely a passive recipient of domesticated information, but an active participant in the construction of liberating knowledge [12].

Within this educational conception, the possibility of empowerment is recognized through dialogicity in the construction of new narratives for comic books, using them as a didactic resource that represents the stories of riverside communities. This is achieved through the knowledge that adolescents acquire in community interactions and contact with elders, as they learn to live in the locality in close connection with nature, the culture produced, and the diversity present in the Amazon [13].

Therefore, this study aims to produce a comic book on menstrual dignity with riverside adolescents from the Amazon, guided by the Paulo Freire’s research itinerary.

## Method

This is a qualitative, participatory study with a methodological focus on the development of an educational technology, guided by Paulo Freire’s research itinerary, which comprises three interconnected stages: thematic investigation, coding/decoding, and critical unveiling. The study was conducted between October and December 2023 at a school located on Caratateua Island, Pará, Brazil, with the participation of 10 adolescents [14].

For the development of the educational technology, a production-construction approach within the context was adopted, ensuring the sociocultural quality of the thematic content and a high level of depth, made possible through the co-creation of the technological instrument. This approach is characterized by the participation of the target audience in all stages of the process, including the identification of themes, the development of content, and the definition of the format and features of the technology. In this way, participants not only share their everyday knowledge but also contribute directly to the development of a material aligned with their experiences, perspectives and sociocultural context [15].

**Fig 1:**
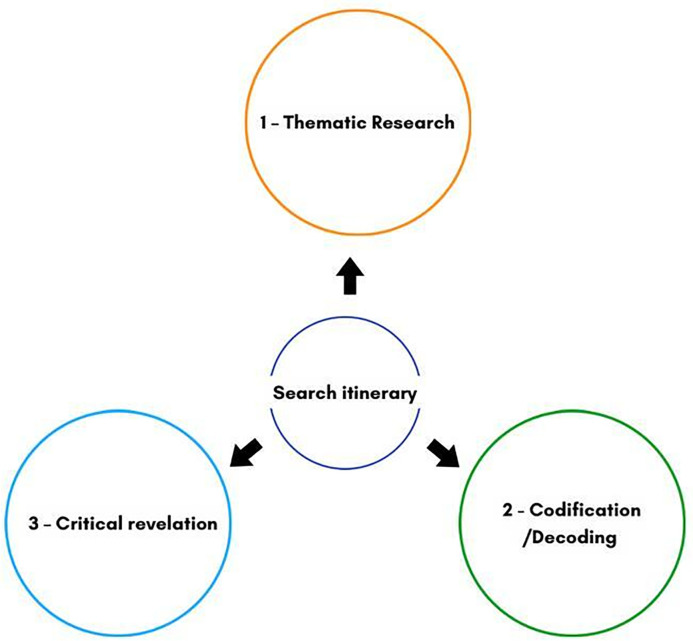
Graphic representation of the stages of Paulo Freire’s research itinerary.

Paulo Freire’s research itinerary represents a qualitative, participatory research approach that prioritizes dialogue in the learning process and has a liberating/emancipatory character ^[^16–18]. It is emphasized that this is not a linear method; the stages of the itinerary occur simultaneously in a back-and-forth process, prioritizing the needs of the participants engaged in the dialogical process, as shown in Fig 1 [19].

**Stage 1: Thematic investigation** – the generating themes related to the study object—menstrual dignity and menstrual precariousness—were identified through the adolescents’ collective reflection on events and health situations in their lives. From this stage, the problematized themes—the generating themes—emerged.

**Stage 2: Coding and decoding** – the generating themes identified in the Thematic Investigation were discussed in Culture Circles, defined as dialogical and participatory group spaces based on Paulo Freire’s approach, in which participants collectively reflect on their experiences and co-construct knowledge. The themes were problematized, contextualized, and reinterpreted from a critical and social perspective on the subject under discussion [20]. Decoding represents the analysis of the situation raised.

**Stage 3: Critical unveiling** – represented the understanding of reality. It was the moment when the action-reflection-action process took place, helping the adolescents to comprehend the raised themes, highlighting the need for concrete action to overcome obstacles and contradictions, as well as to achieve a better quality of life [19–21].

Initial contact was made with the school principal to present the research and to identify the location for data collection, as well as to locate potential research participants. On this occasion, the invitation was formally extended, and information was provided regarding the research objectives, Paulo Freire’s research itinerary, guidelines on participation, and ethical considerations. The administration was consulted about the best times for meetings to collect data, and was informed about the risks and benefits, as well as the signing of the Informed Consent Form.

The study was conducted at a school located on Caratateua Island, which serves students from Early Childhood Education, Elementary School, Youth and Adult Education, and Technical Vocational High School, with Environmental Education as its guiding pedagogical practice.

The participants were 10 adolescent girls in elementary school, corresponding to stages ranging from pre- to post-menarche. This educational cycle refers to middle elementary school (6th to 9th grade). Participants were selected using the “snowball” sampling technique. Through the school administration, parents of adolescents present at the school during a school-organized event (internal sports activities) were informed about the research and asked to provide authorization for their daughters to participate, via the signing of the Informed Consent Form and the Assent Form. A total of 15 adolescents were invited, however only 10 attended the first Culture Circle and were therefore included in the study, with no additional recruitment after this stage.

Adolescents were selected based on inclusion criteria: female, aged between 12 and 15 years, enrolled at the school, attending morning classes, and residing on Caratateua Island. Adolescents who were absent on the date of the first meeting or who missed at least two Culture Circle sessions were excluded.

Six Culture Circles were held, distributed as follows: two during the thematic investigation, two during coding/decoding, and two during critical unveiling. Each session lasted approximately 1.5 to 2.5 hours and was facilitated by the researcher, involving group discussions, interactive activities, and the use of visual and written materials to stimulate reflection and dialogue among participants.

All 10 adolescents attended the first and second sessions. Participation decreased in subsequent meetings, with six adolescents attending the third session and four adolescents attending the fourth, fifth, and sixth sessions. This number was sufficient for the adolescents to understand the meanings and to propose reinterpretations that enabled the production of the educational technology.

The meetings took place at the school itself, in one of the pedagogical coordination rooms. The schedule of dates and times was collectively established during the first meeting and adjusted in subsequent sessions based on the school routine; therefore, the weekly intervals between meetings varied slightly.

These intervals are justified for several reasons: to allow participants time to reflect on the discussions from each meeting so they could come to the next with new proposals, ideas, and needs to be addressed collectively, as well as to allow the researcher-mediator to organize the data produced at each meeting and provide feedback at the following session.

The Culture Circles were audio-recorded, and the materials produced were photographed to ensure the most accurate documentation of group discussions and constructions in the field diary. This diary consisted of statements verbalized by the participants or observed by the researcher, which contributed to the data analysis and the development of the study findings.

**Fig 2:**
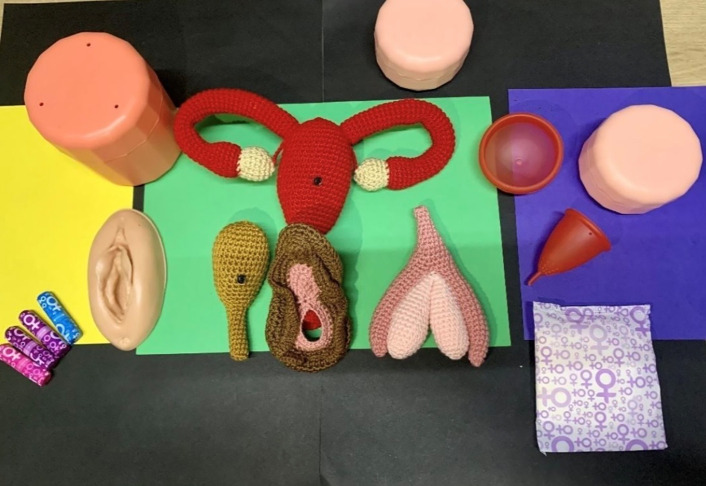
Educational materials used during Culture Circle sessions, including crochet anatomical models and menstrual products.

Each Culture Circle was organized to guarantee the participation of the adolescents, using various resources (images, videos, texts, drawings, paintings, illustrations, crochet materials) to enhance participant engagement (Fig 2).

The Culture Circles were audio-recorded, and the materials produced were photographed to document group discussions and collective constructions. Field diary notes were also maintained to support data organization and contextualization.

Audio recordings were transcribed verbatim, with the omission of any identifying information. The data corpus, composed of transcripts, field notes, and visual materials, was analyzed using inductive thematic analysis aligned with Paulo Freire’s research itinerary. Coding was conducted by the main researcher through iterative reading and refinement of themes, based on recurring patterns in the data. To enhance analytical rigor, the findings were discussed with the research team.

The number of circles was determined based on the principle of theoretical saturation of content. The quality of the information is directly linked to the sensitivity and degree of divergence of each piece of information or content addressed. Theoretical saturation was reached when a certain redundancy or repetition of content emerged in the discussions, as well as when consensus was established regarding the content and appearance of the educational technology.

The study was approved by the Research Ethics Committee of the Magalhães Barata School of Nursing at the State University of Pará, Brazil, under CAAE number: 68147923.3.0000.5170. All participants had authorization from their legal guardians through the signing of the Informed Consent Form and the Assent Form. They were all provided with explanations regarding the objectives, importance, and contributions of this research, and were informed of their right to withdraw from the study at any time, as well as the confidentiality of their identities. Participants were identified by the alphanumeric codes A1, A2, A3.

The product was developed through a collective, participatory, and democratic process during the Culture Circle sessions, with the active involvement of adolescents in decision-making, while the researcher acted as a facilitator, supporting the organization and refinement of ideas. Participants defined the format of the educational technology as a comic book and contributed to the development of its content and narrative structure, based on themes common to their experiences and discussions, collaboratively creating the storyline, characters, and dialogues. Thus, the final product reflects the adolescents’ perspectives and lived experiences regarding menstrual dignity.

## Results

Ten adolescents formed the group for the sessions. Five participants were twelve years old, two were thirteen, two were fourteen, and one was fifteen years old. The ten participants were enrolled in three different school grades: two in the 6th grade, six in the 7th grade, and two in the 8th grade.

In the first Culture Circle, the investigation of generating themes took place, initiating reflection on the adolescents’ experiences and situations related to menstruation in their daily lives. At this point, the generating theme was identified along with a problem-situation—in this case, the menstrual reality of riverside adolescents on Caratateua Island—which was used to trigger discussions in the Culture Circles about the situation and the problems they were experiencing.

The highlighted themes fostered discussion by encouraging participants to share personal experiences, express doubts, and compare perspectives, opening space for other related topics. These conversations led to new perceptions and reflections about the adolescents’ lives. In this context, the researcher-mediator facilitated the connection between the participants’ personal experiences, society, and their cultural environment. Thus, this stage is marked by discovery and the investigation of key themes arising from the daily lives of the adolescents participating in the Culture Circles. It is from this stage that the themes to be problematized emerged.

### First Culture Circle – Introduction to the theme “Menstruation”

The activity began with the introduction of the participants: “Who am I, where do I come from, what grade am I in?” This was followed by a group activity focused on identifying similarities and strengthening bonds among everyone. In pairs or trios, the adolescents were invited to identify and write down on a sheet of paper which characteristics they shared and that brought them together, based on the following questions: “How do I see menstruation in my life today?” “Do I stop doing anything during menstruation?” “Do I have someone to talk to about my menstruation?”

Using the available materials—sheets of paper and colored pens—the questions were answered through drawings, phrases, and short statements. The responses included views of menstruation as something normal; that they have everything they need during their menstrual period (pads with wings, water, medicine for cramps, chocolate, comfortable clothing); and that they rely on family support, especially from their mothers, to talk about menstruation.

Some difficulty was observed in carrying out the proposed activities, with frequent requests for examples to help understand the tasks. Additionally, side conversations were common, often unrelated to the topic being addressed.

Based on the experiences shared during the first circle, a difficulty was observed in the adolescents’ ability to reflect on and verbalize their thoughts about menstruation, particularly when they were asked to express their opinions on the topic, despite it being a recurring monthly experience.

At this initial stage, the menstrual period was associated with negative feelings, such as anger and sadness, which affect daily routines and interpersonal relationships with family members. The participants reported that these feelings are sometimes alleviated by consuming sweets. When expressing their feelings and perceptions regarding menstruation, the adolescents stated the following:

*“So, I don’t really like it, because I feel a lot of things—lots of anger, I get emotional, also have some anger outbursts, a strong craving for sweets, but the desire to lose weight is stronger, [...] yeah, so what I don’t do is, I avoid exercising so it doesn’t come too much.” (A1)*

*“Menstruation is really bad, because it makes you want to hit everyone and we get sensitive, because when people say things that don’t make sense to us, we get very sad and take it to heart.” (A6)*

*“Oh, about menstruation, we said we don’t like it, but we accept it, [...] and when going out we avoid the beach and the pool. In her case (A10), she goes to the gym and prefers not to go on those days, so that’s it.” (A5)*

*“[…] We don’t go swimming in the pool or beach, we avoid leaving the house [...] I eat a lot of sweets, a lot of junk, to the point that my mom scolds me, and when I’m in a lot of pain, I throw myself on the floor or play around with my nephew. I avoid eating greasy food and eggs, because they say it causes a bad smell.” (A4)*

The statements highlight changes in the adolescents’ routines during their menstrual period, with adjustments in daily activities due to menstrual flow and cramps. Cultural beliefs regarding dietary restrictions were also mentioned, especially concerning so-called “remoso” foods, such as eggs, which are associated with undesirable body odors. Regarding physical activity, some adolescents reported avoiding it to prevent increased bleeding, while others continued with their usual activities, such as playing soccer, running, and dancing.

As for swimming in the sea or pool—activities that are common in the adolescents’ daily lives—there was unanimous agreement to avoid them during menstruation, due to the fear of blood leaking into the water. This topic sparked questions and reflections during the discussion, particularly concerning the use of internal menstrual products (tampons), as illustrated by the following statements:

*“They say you can’t, because it will leak.” (A6)*

*“But there’s that OB (tampon), right?” (A4)*

*“There’s no way to go because the blood will leak into the water. They say that because I’m very young, I shouldn’t use a tampon.” (A1)*

The adolescents reported feelings of loneliness and difficulty in sharing doubts and experiences about menstruation. The vast majority expressed having no one to talk to about the subject, considering it too intimate to discuss with family members or others. When such exchanges did occur, they were uncomfortable moments often accompanied by regret. Within the school context, few felt comfortable addressing the topic, occasionally turning to the coordinator but not perceiving the environment as welcoming. The following statements highlight this reality:

*“So, I try to avoid it as much as possible because I don’t like it, I don’t like it, I don’t have that much intimacy to talk about it with relatives.” (A1)*

*“I think the worst part of menstruation is not having anyone to talk to. I think when menstruation comes, it’s very stressful (laughs).” (A8)*

*“[...] I have someone to talk to, and she doesn’t, like my best friend—I can open up to him.” (A2)*

These statements reinforce the importance of dialogical, welcoming, and safe spaces for listening to and sharing experiences related to menstruation, promoting the exercise of autonomy and strengthening menstrual dignity.

Subsequently, questions began to emerge spontaneously during the conversation. The researcher-mediator then proposed that each participant write down their questions on available post-it notes, which were placed on a sheet in the center of the table to be discussed collectively. Among the questions raised by the adolescents were: Why do mood changes occur? What do menstrual cramps mean? Why do breasts hurt during menstruation? Why do women menstruate? What is the purpose of the uterus and why does it contract? Does using a tampon (internal menstrual product) cause loss of virginity?.

The adolescents continued to verbalize their doubts among themselves spontaneously, sharing opinions based on their knowledge and experiences, which helped them identify similar situations among themselves, strengthening group bonds and fostering a space for listening, exchange, and collective recognition.

**Fig 3:**
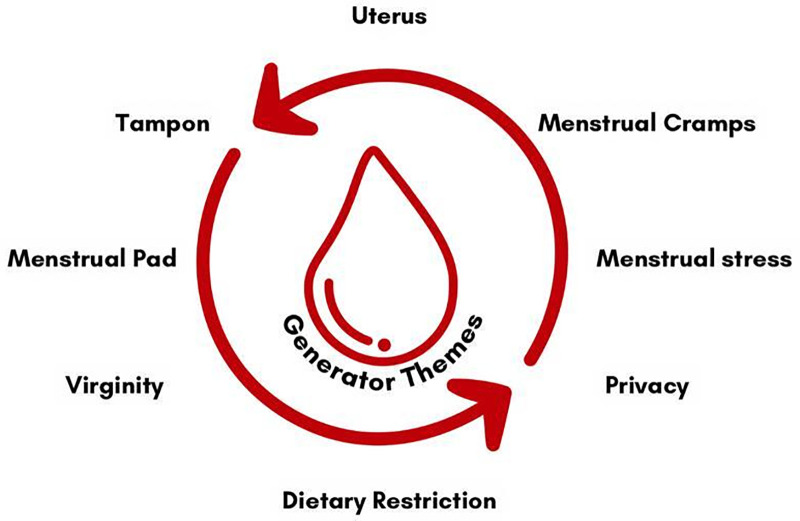
Generating themes emerged from the first meeting.

At the end of this first meeting, the participants’ interest in deepening the topic of menstruation became evident, motivated by the generating themes that emerged throughout the activity (Fig 3), as well as by the doubts shared and discussed in search of collective understanding. The creation of a space for active listening and shared knowledge fostered a critical problematization of the menstrual experience, aligned with Freire’s proposal of liberating dialogue.

This first meeting concluded with a warm “see you soon,” reaffirming the commitment and the expectation of reunion at the next circle.

### Second Culture Circle – Paths to menstrual knowledge

The meeting began with the validation of discussions and records from the previous circle, highlighting similarities and differences in the adolescents’ experiences and reinforcing the importance of conversation. The Culture Circles methodology was presented with an emphasis on the participatory construction of knowledge about menstruation, aimed at producing an educational technology intended for the participating adolescents and other girls from similar contexts.

As a conversation starter, the video *Papo Calcinha – Dubbed Comic* [22] was shown, which addresses menstruation in a playful and accessible way. Then, the motivating question was posed: “Which menstrual rights do I exercise?”, provoking critical reflections on the menstrual cycle and the recognition of rights based on the experiences shared by the adolescents.

After the video screening, the adolescents did not identify with the character presented, revealing difficulties in reflecting on their own menstrual experience. To stimulate the conversation, the researcher-mediator posed complementary questions: What type of sanitary pad do you use? Is it uncomfortable or comfortable? How many times a day do you change the pad? Do you have access to water for menstrual hygiene? The reflective mediation proved effective in promoting engagement in the discussion.

Regarding the types of pads, all reported using disposable pads, although they demonstrated knowledge of other types, such as overnight disposable pads, with/without wings, and internal pads (tampons). Two generating themes were most discussed: menstruation at school and the first menstruation, as these are delicate experiences that require greater attention in menstrual care, marked by feelings of insecurity and shame.

Although no situations of precariousness were identified regarding basic menstrual hygiene management items, gaps in menstrual education were observed. At the end of the meeting, the group collectively defined the generating themes to continue the discussion: types of pads, cramps, menstrual cycle functioning, and nutrition during the menstrual period. The adolescents committed to seeking new information to support the next stage of the process—coding, understood as the stage in which participants transform their experiences and acquired knowledge into representations through drawings, phrases, or situations to support collective reflection.

From the initial analysis of the statements, conducted collaboratively with participants during the second Culture Circle and mediated by the researcher, expressions representative of the group’s experiences emerged, such as: “I’m embarrassed to ask for pads,” “if it happens at school, I put on a jacket or backpack,” “when I have doubts, I search on Google,” “I have no one to talk to,” “it’s cool to become a young woman, it means we’re growing”.

### Third Culture Circle – Reflection on menstrual dignity

**Fig 4:**
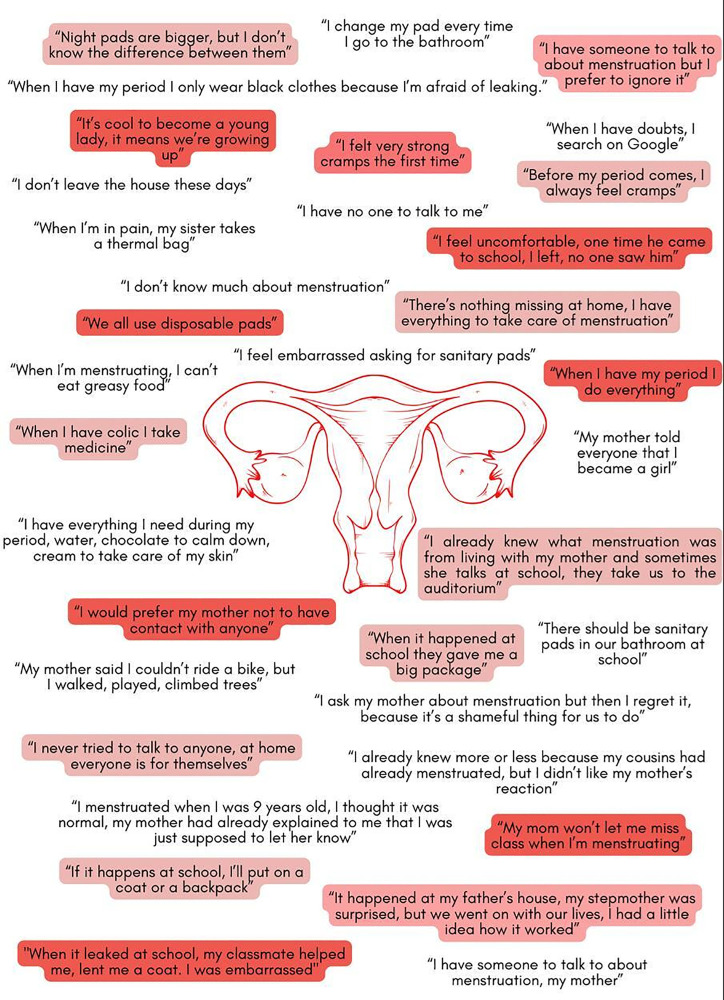
Synthesis of Key Ideas derived from adolescents’ statements, organized into cards during the coding stage and collectively validated in the third Culture Circle session.

The meeting began with the presentation of approximately 35 key ideas displayed on cards, previously extracted from the adolescents’ statements through researcher analysis (Fig 4). After a brief review, participants collectively confirmed that the cards accurately represented the content discussed in previous sessions. Participants were then given approximately five minutes to individually examine the material, followed by a reflection guided by three questions: Which situations presented on the panel do you identify with the most and why? What could be changed and how? What knowledge do you believe you have about menstrual dignity and/or the generating themes presented? How could you expand this knowledge?

The discussion was enriched with the presentation of alternative menstrual products, such as menstrual cups and internal pads (tampons), demonstrated with the help of crochet models. The physical representation of these items generated curiosity and excitement, especially regarding the use of tampons and their relationship to virginity. This is a concern frequently reinforced by family members. The researcher-moderator welcomed the questions and clarified, in a didactic manner, the anatomical diversity of the hymen, deconstructing myths associated with the topic.

Next, small excerpts from texts, articles, and booklets about menstrual dignity were distributed, initiating the theorization stage. From this strategy, critical reflections and greater participation from the adolescents emerged, as they began to relate their experiences to a broader understanding of the menstruation phenomenon, fostering the processes of decoding and unveiling possibilities for transformation in their daily contexts.

**Fig 5:**
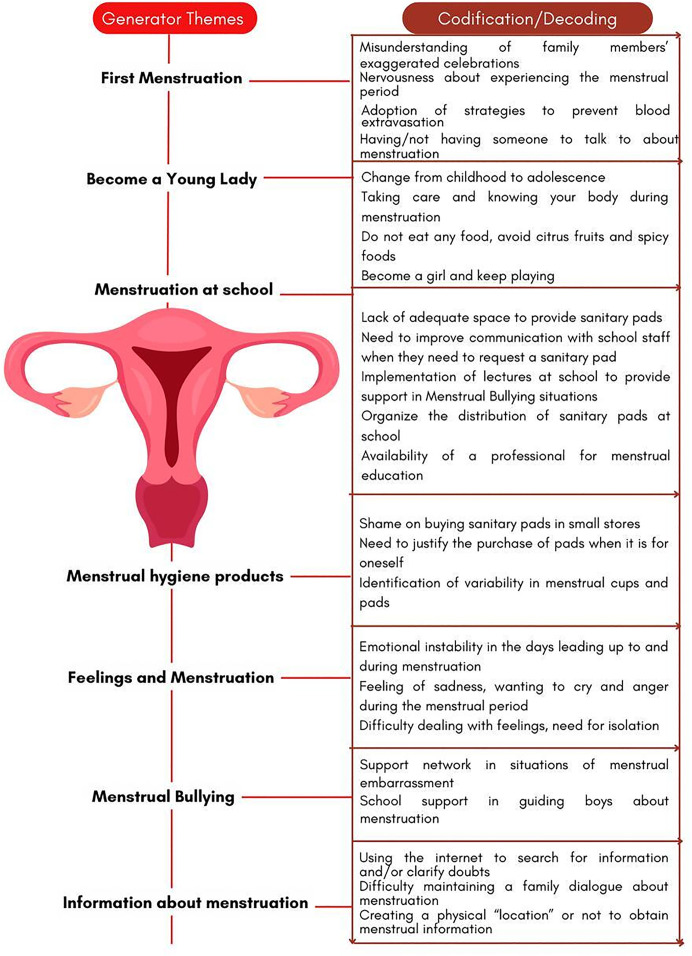
Synthesis of generating themes derived from adolescents’ statements, grouped and explored through coding and decoding during the third Culture Circle.

In this context, seven generating themes were grouped: menstruation at school, first menstruation, menstrual hygiene products, feelings and menstruation, menstrual bullying, “becoming a young woman,” and menstrual information and hygiene, which were explored through the processes of coding and decoding (Fig 5).

### Fourth Culture Circle – Critical unveiling and menstrual empowerment

This meeting focused on the stage of critical unveiling proposed by Freire, providing the adolescents with the opportunity to reflect on their menstrual experiences and possibilities for transforming their reality, linked to the process of re-signifying menstruation and the potential empowerment for promoting menstrual dignity in their context.

An excerpt from the video “*Menstrual Poverty – The Film*” was used to introduce the debate, raising awareness among the group about the impacts of menstrual poverty and encouraging discussions on coping strategies and the role of adolescents as agents of change.

Based on the discussions and reflections, the process of planning the educational technology was initiated. Four stages were conducted: defining the format, content, characters, and narrative approach.

In the first stage—defining the format of the educational technology—participants were presented with two initial options: producing a short video or developing illustrated content in the form of a comic book. After group discussion, the adolescents voted for the comic book format, justified by its accessibility, appeal, and the possibility of both printed and digital distribution.

In the second stage, participants collectively defined the content to be included in the educational technology through group discussion. To support this process, they revisited a video presented during the second Culture Circle, which helped stimulate ideas and reflections. The adolescents expressed concern about not reproducing an existing storyline and, therefore, proposed modifications to create an original narrative aligned with their own menstrual experiences, particularly within the school context.

The group selected the following content: access to clean water; access to disposable and reusable menstrual pads; menstrual dignity; menstrual bullying; and family dialogue about menstruation.

In the third stage, the characters to be included in the educational technology were defined: the main character, two elderly people (grandparents), two school friends, and the school coordinator. This stage required reflection on all the steps needed to outline the story.

Elements such as physical features, age, personality, culture, socioeconomic conditions, and family relationships were all developed and incorporated into the story. For example, the red streak in the main character’s hair refers to menstruation, and the school group was named “Peixe-Boi” (Manatee), considering the symbolic function of animal references, as is common among the participating adolescents. The character’s age was defined based on the most common age among participants.

In the fourth stage, the narrative was built: the main character is an adolescent girl living on Caratateua Island who experiences her menarche in a context of vulnerability, faces embarrassment, identifies her support network, and begins a process of empowerment. Elements such as the red hair streak and the class name “Peixe-Boi” were incorporated to reinforce the group’s local and cultural identity.

### Fifth and sixth Culture Circles – Creation of the educational technology

In the last two meetings, the creation of the educational comic book began. The script was systematized, and the scenes of the comic were detailed, focusing on the main character, who lives in a situation of socioeconomic vulnerability and menstrual insecurity — a reality similar to that of some adolescents from Caratateua Island. Although participants reported access to menstrual products, the construction of this narrative was supported by processes of collective reflection and the problematization of realities observed in their social contexts, further expanded through the educational materials presented throughout the sessions.

The participants envisioned the settings, dialogues, and emotions experienced, especially in relation to the grandmother and friends, who were considered important sources of support and information exchange about menstruation.

The proposal was based on a collective construction of the narrative, grounded in real experiences. As suggested by one of the participants:

*“We can turn all of this into a story where the girl, in this case her, started menstruating early. Her family doesn’t have many resources, no money to buy [products]... [...] It would be really cool.” (A1)*

Menstrual precariousness was addressed critically, highlighting the use of improvised cloths and the scarcity of basic resources such as water:

*“Like, then the grandmother gets a cloth for her [...] the grandmother has a burst of joy and she doesn’t like it.” (A1)*

*“The water came once and didn’t come once [...].” (A4)*

Despite the challenges, the character demonstrates resilience and autonomy, as in the episode where she decides to ride a bicycle, going against her grandmother’s belief:

*“Her grandmother says she doesn’t ride a bicycle, but she was stubborn and went secretly.” (A7)*

The adolescents also included situations of menstrual bullying, emphasizing the importance of support and solidarity among peers:

*“We have to show the boys too that it’s a normal situation.” (A7)*

*“And then they passed [it] on a sweatshirt. [...] Then he takes it off and gives it to her.” (A4)*

Throughout the story, transformative elements were introduced, such as institutional support from the school, educational activities, and collective actions advocating for menstrual dignity. In addition, participants reflected on the potential impact of disseminating the comic book, particularly through digital platforms, as a way to reach broader audiences and promote social change:

*“Protest. Like, there was a protest and stuff. [...] The coordinator helped.” (A1)*

*“In the end, I think... she could talk to the coordinator [...] Then there was another lecture.” (A2)*

*“Since we are going to put it on the internet, it will reach the politicians who will also help those who don’t have access.” (A4)*

Elements such as the cover, title, and aesthetic composition were developed based on the ideas discussed in the circles. The cover was set in the school’s Pedagogical Support Center, and the chosen title, “Menstruation: A Struggle for Menstrual Dignity,” symbolizes the character’s journey in pursuit of her rights and the importance of youth empowerment regarding the topic.

The process of creating the comic book also functioned as a pedagogical and affective tool. The adolescents reported significant changes in their perceptions about the subject and about themselves:

*“Here I talked about many things I had never talked about before [...]. This menstruation thing has many aspects, so it’s important to talk about it at school too.” (A2)*

*“It was nice to participate in your research; at first, it’s kind of difficult to talk about these topics, but then we get comfortable.” (A1)*

*“I had never participated in something like this before, where our story will really come to life [...] it was us who thought and decided.” (A7)*

*“The part about creating the story and the characters was the best [...], I can’t wait to see it finished.” (A4)*

**Fig 6:**
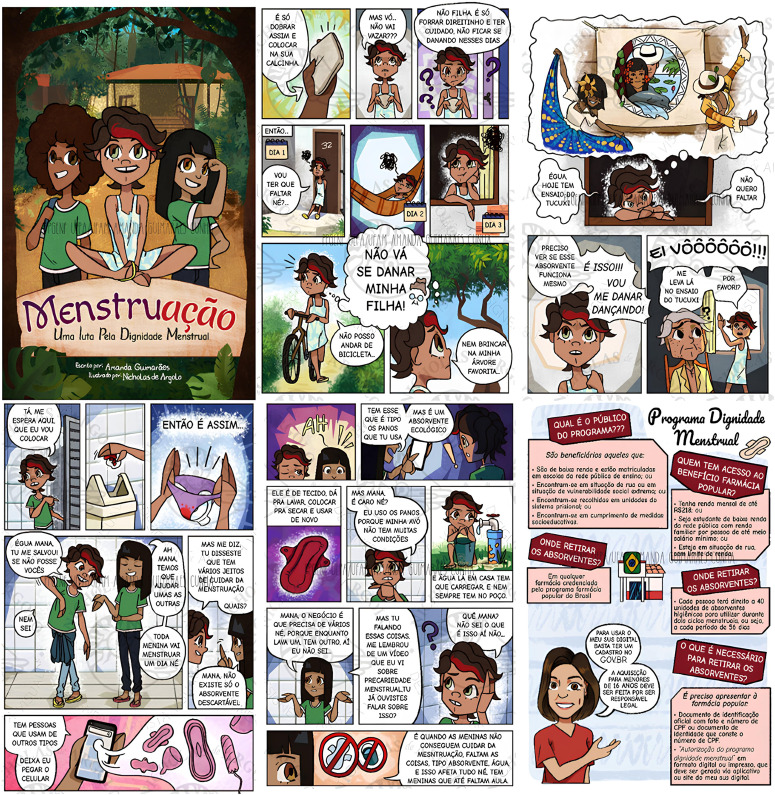
Excerpt from the comic book on menstrual dignity.

The comic book, aimed at adolescents aged 12 and up, was designed as a tool for awareness and education, and can be printed and distributed in schools and communities. The illustration process was carried out by a local artist, highlighting elements of Amazonian culture, such as the tree-lined school setting on Caratateua Island and the red streak in the character’s hair, a symbol of menstrual empowerment (Fig 6).

## Discussion

By organizing the adolescents in a circle, without hierarchy, listening to each other and engaging in dialogue, contact with different perspectives is fostered, reflection on the ongoing experience is deepened, and through this emancipatory process, all learn from both individual and collective realities, thereby expanding their repertoires for menstrual health [23].

From the Freire’s perspective, understanding the reality and context of the learners is fundamental for them to make sense of their reality in order to liberate themselves. The exchange and sharing of experiences and information between learner and educator occurs through a process that enables the construction of knowledge meaningful to the learner. Thus, the learner becomes not only an agent who receives domesticated information but also someone who constructs liberating knowledge [12].

Thus, the adolescent audience requires dialogical spaces to understand themselves amid the transformations of adolescence, considering that the autonomy and emancipation fostered by the culture circle highlight Freirean principles by recovering concepts such as the relevance of research for unveiling the world of adolescents and empowerment through knowledge [24].

From this perspective, the transition from adolescence to adulthood among girls has been described in the literature as sometimes associated with distressing experiences related to limited knowledge about menstruation. The menstrual cycle and reproductive health are sensitive subjects and, therefore, can be influenced by stigma and taboo, or even by socially acceptable responses, considering the adolescent age group. The findings of this study highlight the importance of gender-sensitive educational strategies at the family, community, and school levels, as well as the need to demystify menstrual concepts in order to address the restrictive practices identified among participants [25,26].

The findings of this study reveal that menstruation is still surrounded by silence, embarrassment, and difficulty in open communication, as evidenced by participants’ reports of discomfort in discussing the topic and the use of indirect expressions. These results suggest that menstruation continues to be socially constructed as something that should be hidden, a phenomenon widely described in the literature. Across different cultural contexts, menstruation is often expressed through euphemisms rather than direct language, reinforcing stigma and limiting open dialogue about the topic [27].

In this context, the reading of sequential narratives used in the comic book emerges as a social, communicational, and historical phenomenon, surrounded by characteristics, structures, and forms demonstrated as a resource to promote reflections on the role of individuals in society and capable of causing significant social impacts. Comics engage readers from various distinct social layers who, by interacting with such media products, are impacted by this universe of images, texts, and contextualizations that evoke realities very close to everyday life [28].

In this study, the development of the comic book enabled adolescents to engage in a reflective process, expressing their experiences and perspectives on menstruation through collective construction. Participants noted the potential of this format to address social issues related to menstrual dignity, such as stigma, access to resources, and communication barriers, particularly within their own social contexts. These findings suggest that the use of sequential narratives may support meaning-making processes and the exploration of everyday experiences, contributing to the problematization of contemporary issues, such as menstrual dignity, and to reflection on broader social aspects, including inequalities, health, education, and living conditions [28].

Regarding the limitations of this study, the difficulty in maintaining the engagement of the adolescents in the proposed activities is considered. Additionally, the cultural and regional specificities demand a careful adaptation of the language and topics addressed to connect with the local reality. Another challenge is the initial resistance to discussing menstruation, which may be associated with taboos and stigmas deeply rooted in these communities.

This study contributes to unveiling the menstrual reality of riverine adolescents, taking into account their socioeconomic conditions and local knowledge about the menstrual cycle, thus supporting the improvement of women’s health care, especially adolescent health care and the promotion of menstrual dignity. Furthermore, the participatory development of the educational technology — a comic book — expands the nurse’s pedagogical strategies as a health educator, providing an accessible and contextualized resource to be used in schools and health units. By integrating scientific and popular knowledge, it proves to be a tool that reinforces transcultural care and proposes a dialogical approach based on Paulo Freire, valuing adolescent autonomy and strengthening emancipatory practices.

## Conclusion

The dialogue established throughout the Culture Circles allowed not only a sensitive listening to the reality of the adolescent participants but also fostered the strengthening of menstrual empowerment, grounded in the recognition of their own bodies as cyclical and in the appreciation of individual and collective experiences. By discussing cultural, dietary, and emotional aspects related to menstruation, rigid and socially imposed silencing patterns were broken, promoting critical awareness and authenticity from a perspective of liberating existing stigmas and taboos.

The experience proved to be a unique opportunity to articulate academic knowledge with the personal experiences of the adolescents, fostering an authentic narrative construction rooted in affective memories, often similar to those of the current generation. This connection enabled a powerful and engaged creative process, contributing to the development of an educational technology that is not only accessible but also truthful and sensitive to the reality of the subjects.

The production of the comic book as an educational technology proved viable and effective as an empathetic and pedagogical tool, capable of demystifying taboos, provoking reflection, and expanding the debate on menstrual dignity. By exploring concrete experiences and subjectivities, the constructed narrative achieved a more diverse, culturally situated, and emotionally significant representation, allowing identification and recognition among peers.

Therefore, this study goes beyond theoretical analysis and materializes as a practical action with the potential to transform the attitudes and behaviors of its readers. By giving voice to the adolescents and involving their experiences in the content construction, the comic book becomes a powerful resource to promote respect, equity, and care regarding menstrual health. Thus, menstrual dignity is consolidated as a right to be defended through dialogue, listening, and emancipatory education.
